# Identifying the Alteration Patterns of Brain Functional Connectivity in Progressive Mild Cognitive Impairment Patients: A Longitudinal Whole-Brain Voxel-Wise Degree Analysis

**DOI:** 10.3389/fnagi.2016.00195

**Published:** 2016-08-17

**Authors:** Yanjia Deng, Kai Liu, Lin Shi, Yi Lei, Peipeng Liang, Kuncheng Li, Winnie C. W. Chu, Defeng Wang for the Alzheimer’s Disease Neuroimaging Initiative

**Affiliations:** ^1^Department of Imaging and Interventional Radiology, The Chinese University of Hong KongShatin, Hong Kong; ^2^Department of Medicine and Therapeutics, The Chinese University of Hong KongShatin, Hong Kong; ^3^Chow Yuk Ho Center of Innovative Technology for Medicine, The Chinese University of Hong KongShatin, Hong Kong; ^4^Department of Radiology, The Second People’s Hospital of ShenzhenShenzhen, China; ^5^Department of Radiology, Xuanwu Hospital, Capital Medical UniversityBeijing, China; ^6^Shenzhen Research Institute, The Chinese University of Hong KongShenzhen, China

**Keywords:** mild cognitive impairment, Alzheimer’s disease, resting-state functional magnetic resonance imaging, functional connectivity, connectome

## Abstract

Patients with mild cognitive impairment (MCI) are at high risk for developing Alzheimer’s disease (AD), while some of them may remain stable over decades. The underlying mechanism is still not fully understood. In this study, we aimed to explore the connectivity differences between progressive MCI (PMCI) and stable MCI (SMCI) individuals on a whole-brain scale and on a voxel-wise basis, and we also aimed to reveal the differential dynamic alteration patterns between these two disease subtypes. The resting-state functional magnetic resonance images of PMCI and SMCI patients at baseline and year-one were obtained from the Alzheimer’s Disease Neuroimaging Initiative dataset, and the progression was determined based on a 3-year follow-up. A whole-brain voxel-wise degree map that was calculated based on graph-theory was constructed for each subject, and then the cross-sectional and longitudinal analyses on the degree maps were performed between PMCI and SMCI patients. In longitudinal analyses, compared with SMCI group, PMCI group showed decreased long-range degree in the left middle occipital/supramarginal gyrus, while the short-range degree was increased in the left supplementary motor area and middle frontal gyrus and decreased in the right middle temporal pole. A significant longitudinal alteration of decreased short-range degree in the right middle occipital was found in PMCI group. Taken together with previous evidence, our current findings may suggest that PMCI, compared with SMCI, might be a “severe” presentation of disease along the AD continuum, and the rapidly reduced degree in the right middle occipital gyrus may have indicative value for the disease progression. Moreover, the cross-sectional comparison results and corresponding receiver-operator characteristic-curves analyses may indicate that the baseline degree difference is not a good predictor of disease progression in MCI patients. Overall, these findings may provide objective evidence and an indicator to characterize the progression-related brain connectivity changes in MCI patients.

## Introduction

Mild cognitive impairment (MCI) causes slight but measurable cognitive impairment that does not influence the activities of an individual’s daily life (Gauthier et al., 2006; Brooks and Loewenstein, 2010). MCI can be considered as an intermediate state between normal cognition and dementia; therefore, patients with MCI are at risk of developing Alzheimer’s disease (AD) or other types of dementia. However, not all MCI patients become demented during their lifetime, and some may remain relatively stable or even improve to normal after a long period of follow-up (Busse et al., 2006). Even among the patients with the conversion from MCI to AD, the speed of progression may be largely divergent. The underlying neural mechanisms responsible for the disease progression from MCI to AD remain to be fully elucidated.

Identifying the neurobiological basis for the progression of MCI may be extremely important and has aroused much scientific interest in recent years. Because it is widely recognized that no therapies are able to stop or reverse the disease except for early intervention before AD occurrence (Sperling et al., 2011), sensitive and objective indicators for the development of AD in MCI patients are required. Previous neuroimaging studies of different modalities (Whitwell et al., 2007; Spreng and Turner, 2013; Lopez et al., 2014; Spalletta et al., 2014; Cerami et al., 2015) have identified that brain anatomical, metabolic and/or functional changes may be involved in the progression of MCI to AD. Comparatively, convergent morphometric results have demonstrated that the middle and inferior temporal gyrus (Whitwell et al., 2007; Karas et al., 2008), anterior and posterior cingulate gyrus (Spulber et al., 2012; Spalletta et al., 2014), parietal lobes (Whitwell et al., 2007; Karas et al., 2008) and frontal lobes (Spulber et al., 2012; Spalletta et al., 2014) are more significantly atrophied in progressive MCI (PMCI) patient brains. Spreng and Turner (2013) reported a remarkable decline of structural covariance in the default mode network (DMN) in the brains of PMCI patients. Significant hypometabolism and altered functional connectivity (FC) have been discovered in the tempo-parietal region and precuneus/posterior cingulate gyrus in PMCI patients by comparing with stable MCI (SMCI) patients using positron emission tomography imaging and magnetoencephalography analysis (Lopez et al., 2014; Cerami et al., 2015). Overall, these findings confirm that PMCI and SMCI are able to be distinguished and characterized at the neurobiological level.

Recently, increasing evidence based on advanced brain connectivity analysis techniques suggests that AD may be among the most classic disconnection syndromes (Jacobs et al., 2013). Therefore, we speculated that the PMCI subjects might have more severe brain connectivity dysfunctions than SMCI subjects. Further, connectivity probes have shown a unique advantage over traditionally segregated metrics (e.g., regional activation in fMRI), especially in terms of sensitivity (Meyer-Lindenberg et al., 2005; Esslinger et al., 2009). Moreover, among the various types of connectivity probes, voxel-wise degree analysis, which is based on graph theory, is one of the most recently emerging tools (Hayasaka and Laurienti, 2010). Compared with traditional connectivity methods where measurement is among and dependent on a series of regions of interest (ROIs), voxel-wise degree analysis calculates the connectivity for each voxel across the whole brain and thus provides a comprehensive, unbiased and spatially detailed representation of brain connectivity. Thus, we believe the whole-brain voxel-wise analysis method could detect the brain connectivity alterations in PMCI subjects with high objectivity and sensitivity. However, to our knowledge, this method has not been applied to investigate the difference in longitudinal brain connectivity evolution between PMCI and SMCI individuals.

Based on previous findings, we hypothesize that certain brain disconnection patterns may provide cues to differentiate the SMCI subjects from those with pronounced progression in a short time period and may also provide a sensitive and objective indicator for the prognosis of Alzheimer’s disease. Therefore, in this study, we have two objectives: (1) To comprehensively detect the progression-related brain connectivity changes at whole-brain level in MCI patients; (2) To evaluate whether the connectivity abnormality of PMCI observed at the baseline could be of some predictive value for disease progression. We hope our findings will help to describe how the brain network evolves differently across time in cases of PMCI and SMCI, which may enrich our understanding of the neurobiological mechanisms of AD progression.

## Materials and Methods

### Subject

Clinical and magnetic resonance imaging (MRI) data of all of the subjects used in this study were obtained from the dataset of the Alzheimer’s Disease Neuroimaging Initiative (ADNI) (adni.loni.usc.edu), which was launched in 2003 as a public-private partnership that was led by Principal Investigator Michael W. Weiner, MD. Briefly, the cognitive level of subjects was determined by combining the memory complaints of patients or their families, Wechsler Memory Scale-Logical Memory II (Wechsler, 1987) results, Mini-Mental State Exam (MMSE) scores, and Clinical Dementia Rating (CDR) scores of subjects. Detailed inclusion and exclusion criteria were found in the ADNI2 and ADNI GO protocol^1^. In the current study, for cross-sectional analyses, an additional inclusion criterion was having the clinical follow-up for at least 3 years (for SMCI subjects) or until the progression occurred (for PMCI subjects). Besides, for longitudinal analyses, all subjects should have two rs-fMRI scans at baseline and year-one, respectively. The included MCI subjects were divided into two groups based on whether they converted to AD during the follow-up. At baseline, there were 35 SMCI (male/female, 18/17; mean age, 71.0 years) and 21 PMCI subjects (male/female, 11/10; mean age, 71.4 years) included. At year-one, there were 15 PMCI (male/female, 8/7; mean age, 71.5 years) and 22 SMCI subjects (male/female, 12/10; mean age, 70.4 years) left due to 19 missing subjects during follow-up (**Table 1**; Supplementary Table 1; Supplementary Figure 1 for more detailed information).

**Table 1:** Demographics and Mini-Mental State Exam (MMSE) scores of subjects for longitudinal analyses.

	PMCI	SMCI	*p* value
Age (±SD)	71.5 ± 6.8	70.4 ± 7.6	0.656
Gender (M/F)	8/7	12/10	0.942
Education	16.0 ± 2.8	16.0 ± 2.5	0.899
mFD_bl	0.27	0.26	0.791
mFD_y1	0.30	0.29	0.845
MMSE score (bl)	27.1 ± 1.64	27.5 ± 1.77	0.58
MMSE score (year 1)	26.3 ± 1.75	28.6 ± 1.68	2.6 × 10^-4^
MMSE score (year 3)^∗^	24.1 ± 3.3	28.0 ± 2.0	3.7 × 10^-4^
MMSE score change (year 1 vs. bl)	-0.8	1.1	0.001^†^
MMSE score change (year 3 vs. bl)	-3	0.5	0.002^†^

*PMCI, progressive mild cognitive impairment; SMCI: stable MCI; MMSE, Mini-Mental State Exam; mFD_bl, mean frame-wise displacement at baseline; mFD_y1, mean frame-wise displacement at year-one; bl, baseline; ^∗^Follow-up did not continue in six PMCI subjects after conversion to AD; Statistical level: *p* < 0.05; ^†^indicates the *p* value of the interaction effect between time and group.*

### Image Acquisition

All of the subjects were examined on 3T Philips MRI scanners. The rs-fMRI was acquired using an echo-planar imaging sequence (EPI) with the following parameters: 140 time points, repetition time (TR) = 3000 ms, echo time (TE) = 30 ms, flip angle = 80°, slice thickness = 3.3 mm, spatial resolution = 3.3 mm × 3.3 mm × 3.3 mm and FOV = 212 mm. Additionally, high-resolution three-dimensional T1-weighted images (3D-T1WIs) were acquired using magnetization-prepared rapid acquisition gradient-echo (MPRAGE) imaging with TR = 6.8 ms, TE = 3.1 ms, 170 slices, flip angle = 9°, slice thickness = 1.2 mm, spatial resolution = 1.2 mm × 1 mm × 1 mm and FOV = 256 mm.

### Data Preprocessing

Data preprocessing was performed by using the Statistical Parametric Mapping software (SPM8)^2^ in accordance with the data processing guideline for rs-fMRI provided by ADNI^3^. The first three image volumes of resting-state data were discarded for signal equilibrium and subject adaptation to the fMRI scanning noise. The remaining 137 images were first corrected for the timing differences between each slice and then corrected for head motion using a six-parameter spatial transformation (images with more than 2.0 mm displacement in any of the x, y, or z directions or 2.0° of any angular motion were removed). Next, the resultant images of each subject were registered to the individual 3D-T1WI and normalized to the MNI space using the DARTEL tool of SPM (resampled into 3 mm × 3 mm × 3 mm cubic voxels). After a manual check of the registration effect, detrending and temporal band-pass filtering (0.01–0.08 Hz) were performed to reduce the linear drift and high-frequency noise, respectively. Finally, the six head motion parameters and parameters of the white matter signal, global mean signal and cerebrospinal fluid signal were used as nuisance variables to regress out their residual effects.

### Whole-Brain Voxel-Wise Connectivity Analysis

The whole-brain voxel-wise measurement of FC, i.e., degree map, was constructed for each subject. First, due to 19 missing subjects during follow-up, the number of included subjects was different in cross-sectional (*n* = 56) and longitudinal (*n* = 37) analyses. To make subsequent analyses more precisely confined in the grey matter (GM) voxels, two binary masks were, respectively, constructed for cross-sectional (*N*_voxel_ = 43157) and longitudinal (*N*_voxel_ = 47756) analyses. Each mask was calculated by thresholding the mean GM probability map (generated in the previous step using 3D-T1WIs) of all subjects in corresponding group with a probability threshold of 0.2. Second, the resting-state FC between two GM voxels was calculated as the Pearson correlation coefficient across the time series, and a pair of voxels above a coefficient threshold was considered functionally connected (Eilam-Stock et al., 2014). Here, the threshold of *r* > 0.3 has been widely used in previous studies (Mutso et al., 2014; Contreras-Rodriguez et al., 2015) and was considered effectively limiting the false positive rate and with a comparatively high sensitivity. In this study, to get robust results, we further extended the threshold from 0.3 to a range of 0.2–0.4 at an interval of 0.05 (i.e., *r* = 0.2, 0.25, 0.3, 0.35, and 0.4), and repeated the degree analyses over this range. Similar threshold range has also been adopted in previous studies (Buckner et al., 2009; Zhuang et al., 2015). On this basis, the degree for a given voxel was defined as the total number of voxels that were functionally connected to it at the corresponding threshold. Third, the long-range degree map and short-range degree map were calculated with the Euclidean distance between each pair of connected voxels being, respectively >75 and ≤75 mm (Liang et al., 2013). Finally, the resultant long- and short-range degree maps of each subject were converted with a *z*-transform (by substracting the global mean degree then dividing the standard deviation) to increase the normality of the data, and then were spatially smoothed with a Gaussian kernel of 8 mm full-width half-maximum (FWHM) before group comparison.

### Statistical Analysis

The two-sample *t*-tests were performed to assess the difference in demographic data (including age, gender, and education level) and MMSE scores between PMCI and SMCI groups (or the Mann–Whitney *U*-test was used if the data followed a non-normal distribution). The statistical significance threshold was set at *p* < 0.05.

Longitudinal analyses of the long-range and short-range degree maps were performed using a factorial design in SPM8 with the two main factors of group (PMCI and SMCI) and time (baseline and year-one), and the analyses were repeated at each correlation threshold from 0.2–0.4. Thus, the main difference between groups and the interaction effect between group and time were, respectively, evaluated (Supplementary Figure 2). Here, the main effect of group evaluates the between-group degree difference that is independent of time, while the interaction effect between time and group shows the difference in longitudinal evolution of degree between PMCIs and SMCIs during the 1-year period. The statistical maps were created using a combined threshold of *p* < 0.01 and a minimal cluster size of 54 voxels, thereby yielding an Alphasim correction threshold of *p* < 0.05. It’s worth mentioning that the head motion effect on FC has been identified and emphasized in recent study (Power et al., 2014). Therefore, we compared the mean frame-wise displacement (mFD) between groups at baseline and year-one, and found no significant difference (*p* = 0.791 and 0.845, respectively). The age of each subject was added as covariate to exclude its confounding effects. For the subsequent correlation analysis between longitudinal change of degree and longitudinal change of MMSE score in 1-year duration, a sphere ROI (radius = 3 mm) centered at the peak coordinate of the cluster with significant interaction effect was drawn. Then, the mean degree value of the ROI across all the correlation thresholds over 0.2–0.4 was calculated for each scan of each subject (i.e., mean degree at baseline and mean degree at year-one, respectively). Finally, the difference of mean degree at year-one minus mean degree at baseline was calculated for each subject, and then correlated with the difference of MMSE score at year-one minus MMSE score at baseline. The Spearman correlation was used with a significance level of *p* < 0.05.

Cross-sectional analyses of the long-range and short-range degree maps between SMCI and PMCI group at baseline were conducted using two-sample *t*-test in SPM8, and the analyses were repeated at each correlation threshold from 0.2 to 0.4 (Supplementary Figure 2). The statistical maps of cross-sectional analyses were created using a combined threshold of *p* < 0.01 and a minimal cluster size of 48 voxels, thereby yielding an Alphasim correction threshold of *p* < 0.05. Similarly, mFD were compared and did not show significant between-group difference (*p* = 0.795). The age of each subject was added as covariate to exclude its confounding effect. For testing the prognostic utility of the degree value of significant brain regions for disease progression, several sphere ROIs (radius = 3 mm) centered at peak coordinates of the cluster with significant between-group differences were drawn. Then, the mean degree value of each ROI across all the correlation thresholds was calculated for each subject. Finally, the receiver-operator characteristic-curves (ROCs) and respective area under the curve (AUC) of the ROIs were evaluated.

## Results

### Demographics of Subjects

The age, gender, and education level were matched between PMCI and SMCI groups (**Table 1**; Supplementary Table 1). The median time from the baseline to conversion of PMCI subjects was 2 years. The mean MMSE scores of PMCI and SMCI groups at 1 year of follow-up (i.e., the time point for the second MR scan) were decreased by 1.9 points and increased by 0.2 points, respectively. At 3 years of follow-up, the mean MMSE scores of PMCI and SMCI groups were decreased by 2.9 points and increased by 0.2 points, respectively (**Table 1**).

### Results of Longitudinal Degree Analyses

#### Degree Differences between PMCI and SMCI Patients

**FIGURE 1:**
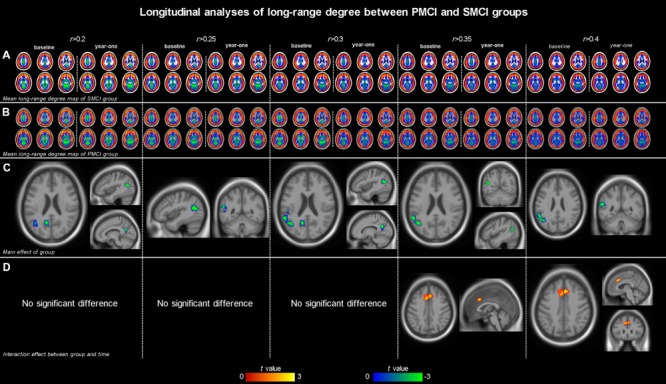
**The mean long-range degree maps of progressive mild cognitive impairment (PMCI) group and stable MCI (SMCI) group and the longitudinal analyses results across the correlation threshold range of 0.2–0.4.** In row **(A,B)**, the mean long-range degree maps of SMCI and PMCI groups at baseline and year-one are shown, respectively. In row **(C)**, the green-blue indicates brain regions with significantly decreased long-range degree in PMCI group compared with SMCI group (controlling for the effect of time). In row **(D)**, the yellow–red indicates brain regions with a significant longitudinal increase of long-range degree in the PMCI group in 1-year duration compared with SMCI group (Statistical level: *p* < 0.01 with a minimal cluster size of 54 voxels, which yields an Alphasim correction threshold of *p* < 0.05).

**FIGURE 2:**
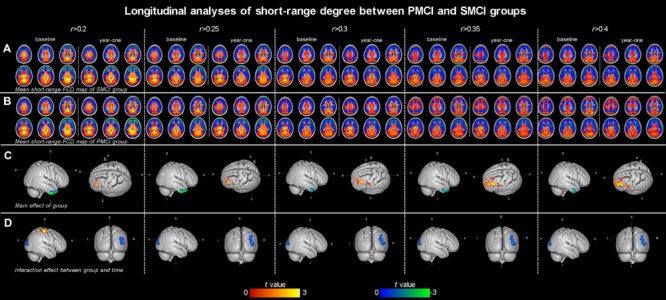
**The mean short-range degree maps of progressive mild cognitive impairment (PMCI) group and stable MCI (SMCI) group and the longitudinal analyses results across the correlation threshold range of 0.2–0.4.** In row **(A,B)**, the mean short-range degree maps of SMCI and PMCI groups at baseline and year-one are shown, respectively. In row **(C)**, the green–blue/red–yellow indicates brain regions with significantly decreased/increased short-range degree in the PMCI group compared with the SMCI group (controlling for the effect of time). In row **(D)**, the green–blue/red–yellow indicates the brain regions with a significant longitudinal decrease/increase of short-range degree in the PMCI group in 1-year duration compared with SMCI group (Statistical level: *p* < 0.01 with a minimal cluster size of 54 voxels, which yields an Alphasim correction threshold of *p* < 0.05).

Both **Figures 1** and **2**; **Table 2** show the main effects of between-group differences (in patients with PMCI vs. SMCI) in the voxel-wise degree, respectively, at the correlation thresholds of *r* > 0.2, *r* > 0.25, *r* > 0.3, *r* > 0.35, and *r* > 0.4. For the long-range degree, a significant decrease was found in the left precuneus gyrus (at the thresholds of *r* > 0.2 and *r* > 0.3) and the left middle occipital gyrus (extending to the supramarginal gyrus, at the thresholds of *r* > 0.2–0.4) in the PMCI group when compared with the SMCI group (**Figure 1C**). For the short-range degree, PMCI patients exhibited decreased degree in the right middle temporal pole (extending to the inferior temporal gyrus, at the thresholds of *r* > 0.2–0.4) and exhibited increased connectivity in the left middle frontal gyrus (at the thresholds of *r* > 0.2–0.4), right supplementary motor area (at the thresholds of *r* > 0.2, *r* > 0.25, *r* > 0.35, and *r* > 0.4) and left precentral gyrus (at the threshold of *r* > 0.3) (**Figure 2C**). Generally, among these significant regions, the left middle occipital gyrus, right middle temporal pole, left middle frontal gyrus, and right supplementary motor area were consistently found with significant degree differences across the correlation threshold range of 0.2–0.4.

**Table 2:** Longitudinal degree analysis between PMCI and SMCI groups by controlling the effect of time.

	AAL region	Correlation threshold (*r* value)	Peak coordinate (MNI)	*t*-value	Cluster size (voxels)
Long-range degree	PMCI < SMCI				
	L precuneus gyrus	0.2	-15 -60 30	-3.2836	53
		0.3	-15 -60 30	-3.4374	56
	L middle occipital/supramarginal gyrus	0.2	-39 -69 15	-4.0591	59
		0.25	-39 -60 15	-4.0421	102
		0.3	-39 -69 15	-4.0191	120
		0.35	-39 -60 15	-4.1288	111
		0.4	-39 -60 15	-4.0635	116
	PMCI > SMCI				
	No significant result was found				
Short-range degree	PMCI < SMCI				
	R middle temporal pole/inferior temporal gyrus	0.2	33 21 -45	-3.6404	139
		0.25	33 21 -45	-3.601	142
		0.3	33 21 -45	-3.6438	76
		0.35	33 21 -45	-3.4172	107
		0.4	33 21 -45	-3.2817	71
	PMCI > SMCI				
	L middle frontal gyrus	0.2	-24 24 45	4.2303	223
		0.25	-36 30 33	4.328	309
		0.3	-39 30 36	3.8274	218
		0.35	-24 24 45	4.4474	471
		0.4	-24 24 45	4.4826	552
	R supplementary motor area	0.2	12 3 54	4.556	53
		0.25	12 6 54	4.6112	56
		0.35	12 6 54	4.5265	57
		0.4	12 6 54	4.3689	53
	L precentral gyrus	0.3	-36 0 33	4.03.4	83

*PMCI, progressive mild cognitive impairment; SMCI, stable MCI; L, left; R, right; Statistical level: *p* < 0.01 with a minimal cluster size of 54 voxels, which yields an Alphasim correction threshold of *p* < 0.05.*

#### Divergent Longitudinal Alteration Patterns of Degree between PMCI and SMCI Patients

The different between-group evolution patterns of degree across the 1-year period were evaluated as significant interactions between the factors of group and time, and the results at the correlation thresholds of *r* > 0.2, *r* > 0.25, *r* > 0.3, *r* > 0.35, and *r* > 0.4 are, respectively, shown in **Table 3**. For the long-range degree, the right middle cingulum gyrus showed significantly increased measurement (at the thresholds of *r* > 0.35 and *r* > 0.4) in PMCI group in the 1-year duration (**Figure 1D**). For the short-range degree, the connectivity in the right middle occipital gyrus significantly decreased (at the thresholds of *r* > 0.2–0.4), while in the right postcentral gyrus significantly increased (at the thresholds of *r* > 0.2) in PMCI subjects (**Figure 2D**) in the 1-year duration. Generally, among these brain regions, only the right middle occipital gyrus was consistently found with significant interaction across the correlation thresholds of *r* > 0.2–0.4. In addition, a significant correlation (*r* = 0.37, *p* = 0.02) was found between the observed degree change in the right middle occipital gyrus with the change of MMSE score in 1-year duration.

**Table 3:** Longitudinal degree analysis on the dynamic alteration pattern between PMCI and SMCI groups in 1-year duration.

	AAL region	Correlation threshold (*r* value)	Peak coordinate (MNI)	*t*-value	Cluster size (voxels)
Long-range degree	R middle cingulum gyrus	0.35	3 21 36	3.4506	54
		0.4	3 21 36	3.4361	54
Short-range degree	R middle occipital gyrus	0.2	36 -87 21	-3.2628	55
		0.25	36 -87 21	-3.2888	67
		0.3	33 -87 18	-3.3403	71
		0.35	33 -87 18	-3.3568	75
		0.4	33 -81 33	-3.339	71
	R postcentral gyrus	0.2	51 -21 57	3.6692	87

*PMCI, progressive mild cognitive impairment; SMCI, stable MCI; L, left; R, right; Statistical level: *p* < 0.01 with a minimal cluster size of 54 voxels, which yields an Alphasim correction threshold of *p* < 0.05.*

### Results of Cross-Sectional Degree Analyses at Baseline

**FIGURE 3:**
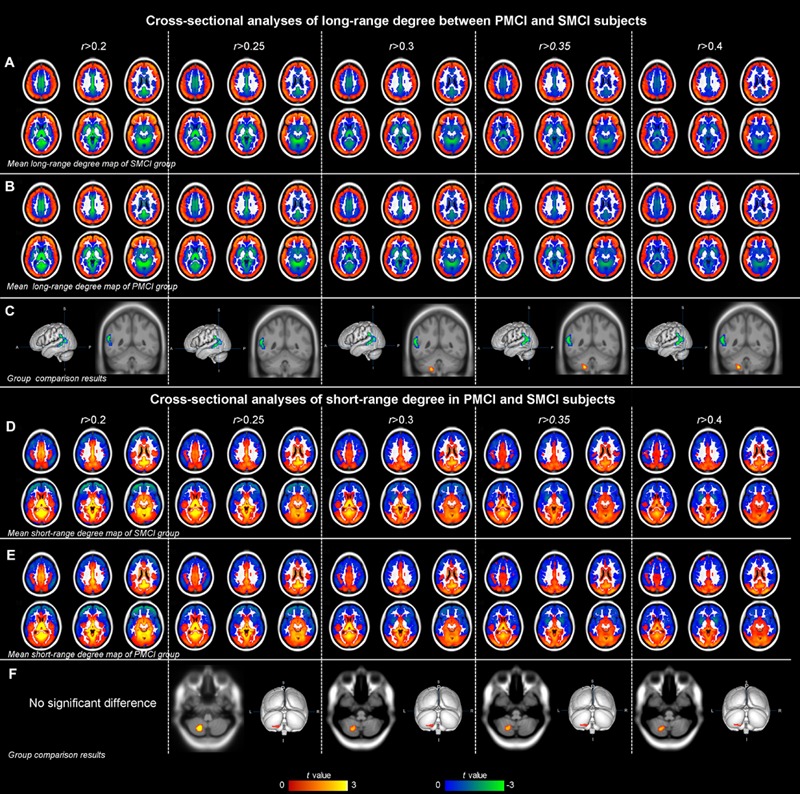
**The mean degree maps of the PMCI group and stable MCI (SMCI) group and baseline cross-sectional analyses results across the correlation threshold range of 0.2–0.4.** The upper panel shows the mean long-range degree maps of SMCI (row **A**) and PMCI (row **B**) groups and the between-group comparison results (row **C**). In row **(C)**, the green–blue/red–yellow indicates the brain regions with significantly decreased/increased long-range degree in the PMCI group compared with the SMCI group at baseline. The lower panel shows the mean short-range degree maps of SMCI (row **D**) and PMCI groups (row **E**) and the between-group comparison results (row **F**). In row **(F)**, the red–yellow indicates the brain regions with significantly increased short-range degree in the PMCI group compared with the SMCI group at baseline. (Statistical level: *p* < 0.01 with a minimal cluster size of 48 voxels, which yields an Alphasim correction threshold of *p* < 0.05).

**Figure 3** and **Table 4** show the cross-sectional differences between SMCI and PMCI groups in long-range and short-range degree at the correlation thresholds of *r* > 0.2, *r* > 0.25, *r* > 0.3, *r* > 0.35, and *r* > 0.4. Compared with SMCI group, consistently decreased long-range degree was located in the left middle temporal gyrus across the correlation thresholds of 0.2–0.4, while consistently increased short-rang degree was found in the left cerebellum in PMCI group across the correlation thresholds of 0.25–0.4.

**Table 4:** Cross-sectional degree comparison between PMCI and SMCI groups.

	AAL region	Correlation threshold (*r* value)	Peak coordinate (MNI)	Cluster size (voxels)	*t*-value
Long-range degree	PMCI < SMCI				
	L middle temporal gyrus	0.2	-57 -57 9	114	-3.4743
		0.25	-57 -51 15	128	-3.4851
		0.3	-60 -51 15	141	-3.572
		0.35	-54 -45 -3	140	-3.714
		0.4	-54 -45 -3	124	-3.7184
	PMCI > SMCI				
	L cerebellum	0.3	-18 -54 -54	79	3.7732
		0.35	-18 -54 -54	91	3.829
		0.4	-18 -54 -54	90	3.7579
Short-range degree	PMCI > SMCI				
	L cerebellum	0.25	-18 -66 -51	49	3.7092
		0.3	-21 -69 -51	53	3.7417
		0.35	-21 -69 -51	57	3.7381
		0.4	-21 -69 -51	58	3.6669
	PMCI < SMCI				
	No significant cluster was found				

*PMCI, progressive mild cognitive impairment; SMCI, stable MCI; L, left; R, right; Statistical level: *p* < 0.01 with a minimal cluster size of 54 voxels, which yields an Alphasim correction threshold of *p* < 0.05.*

**FIGURE 4:**
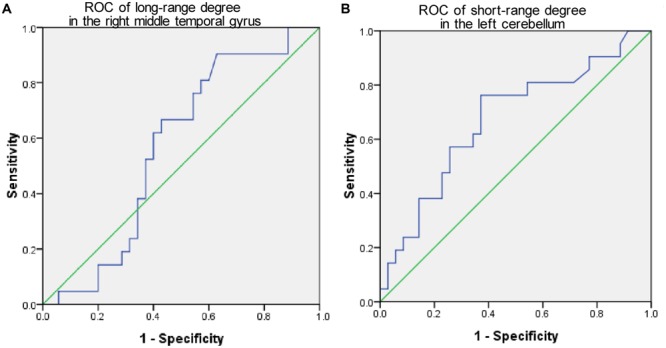
**The Receiver-operator characteristic-curves (ROCs) based on the degree value of brain regions with significant cross-sectional difference between PMCI and stable MCI (SMCI) groups at baseline. (A)** The ROC of the long-range degree in the right middle temporal gyrus; **(B)** The ROC of the short-range degree in the left cerebellum.

The mean long/short-range degree values of the ROIs from the above two significant brain regions were calculated across their respective significant threshold ranges and were used for ROC analyses (**Figure 4**). The short-range degree in the left cerebellum was found with significant AUC value (0.67, with the 95% confidence interval of 0.52–0.82, *p* = 0.03) (**Figure 4B**). However, the AUC value of the left middle temporal gyrus was insignificant (0.57, with the 95% confidence interval of 0.41–0.72, *p* = 0.40) (**Figure 4A**).

## Discussion

In this study, using a comparatively advanced connectivity probe of voxel-wise degree analysis, we comprehensively assessed the connectivity character and its longitudinal alteration pattern across the whole brain in MCI patients with a rapid progression. The longitudinal analysis revealed different dynamic alteration pattern of brain connectivity between PMCI and SMCI subjects in 1-year duration. On the other hand, the cross-sectional comparison between SMCI and PMCI groups identified two brain regions with significant connectivity difference at baseline. However, the ROC analyses results indicated a limited prognostic value of the observed baseline degree difference for disease progression.

Regarding several technical considerations, first, because the low repeatability of rs-fMRI has aroused much concern, we conducted a longitudinal analysis with repeated MR scans in this study. Such a longitudinal design will not only help to limit the low repeatability issue, but also provide a way to observe the dynamic connectivity changes during disease progression. Moreover, combining with the cross-sectional analysis, an evaluation of the predictive value of baseline resting-state FC for disease progression can also be realized. Second, in our previous study (Li et al., 2016), the connectivity measurement of strength was used to evaluate the cross-sectional connectivity difference between PMCI and SMCI. Therefore, in the current study, we adopted another connectivity probe of degree to provide more comprehensive evidence for characterizing the alteration patterns of brain connectivity associated with disease progression in MCI patients. However, it should be noted that the measurement of degree is highly dependent on the selection of correlation coefficient threshold. Therefore, to get robust results in this study, we performed the analyses across a range of correlation coefficient thresholds (Buckner et al., 2009; Zhuang et al., 2015). Third, it is worth noting that parts of the results might change across the threshold range. These inconsistent results are possibly be influenced by the threshold variation and show low robustness. Thus, in current study, we only focused on the consistent results with high robustness, i.e., brain regions that were consistently found with statistical significance across the threshold range. These findings should be considered to reflect the effect of the disease pathology instead of a consequence of parameter changes.

For the longitudinal analyses, three features of the brain connectivity alteration can be summarized. First, for the direction of the between-group FC difference, the long-range FC of PMCI patients can be characterized by a decrease in the significant regions, while the short-range FC may be more variable with either an increase or decrease. The currently identified opposite changing pattern between the long-range and short-range connectivity in PMCI may be similar to the findings in AD patients compared with normal subjects. For example, (Dai et al., 2015) compared FC of whole-brain networks between AD patients and normal controls and found more prominent disruptions in long-range connections. Further, studies based on graph theory consistently have found prolonged path length and stable or increased intra-modular connectivity (see Tijms et al., 2013 for review) in AD brains, suggesting a connectivity organization pattern of decreased integration combined with increased segregation in the AD brain network (Ciftci, 2011). It has been considered that long-range connections link remote brain regions in the network (Achard et al., 2006) and are responsible for information integration to support human cognitive function (Bullmore and Sporns, 2012). Therefore, taken together, we speculate that the decreased long-range connections of PMCI subjects might not only reflect a severe neural impairment pattern of brain network compared to SMCI subjects, but may also underlay a higher risk of progression of the disease. Moreover, higher interaction among neighboring brain regions was noted in previous AD researches and interpreted as a compensatory mechanism (Supekar et al., 2008; Cha et al., 2013). Thus, similarly, the increased short-range connectivity in PMCI perhaps suggests an adaptive response to long-range disconnectivity. However, the specific relationship between decreased long-range and increased short-range connectivity still requires further investigation (Tijms et al., 2013).

For the anatomical distribution of the statistically significant brain regions, not only the classic AD-relevant areas, i.e., memory network (including the middle temporal gyrus, middle frontal gyrus and supramarginal gyrus), but also the sensory-motor areas (including the supplementary motor area) and visual perceptual areas (including the inferior temporal gyrus and middle occipital gyrus) were involved in PMCI subjects. The findings in the memory network are largely in agreement with the susceptible brain regions under AD pathology (O’Connor et al., 2010; Agosta et al., 2012; Cha et al., 2013). For example, disruption of the long-range connections in the left supramarginal gyrus as well as impaired short-range connections in the right middle temporal gyrus of PMCI brains are both consistent with Damoiseaux’s and Agosta’s findings (Agosta et al., 2012; Damoiseaux et al., 2012) wherein disrupted connections were found in these two regions in AD patients and progressed after a follow-up period; however, these abnormalities were not evident in amnestic MCI subjects. Therefore, these overlaps between findings in PMCI and AD may also suggest that compared with SMCI, PMCI presented severe decline of brain connectivity among the AD spectrum of diseases.

In addition to the classic AD-relevant areas, the brain regions with significant connectivity alteration was found in the sensory-motor and visual perception system in the PMCI group. A previous study has confirmed that higher FC of the supplementary motor area in AD patients is associated with poorer cognitive behaviors, e.g., memory function (Adriaanse et al., 2014). Thus, the increase of sensory-motor regions currently identified in PMCI brains in comparison to SMCI brains may represent a higher disease severity. On the other hand, there was a significant decrease in short-range degree in the right inferior temporal gyrus of PMCI patients. There was also a significant decrease in short-range degree in the right middle occipital gyrus of PMCI patients during the 1-year follow-up. These two regions are considered to be the key regions for visual perception (Grill-Spector and Malach, 2004) and have close connections with the sensory-motor system and DMN network (Lerner et al., 2001; Pascual et al., 2015). Therefore, these findings may imply that the disruption of visual cortical connectivity could be of indicative/predictive value for disease progression and may help to explain the visual perception dysfunction that occurs during disease progression in AD/MCI patients (Adlington et al., 2009).

For longitudinal alteration differences (i.e., the interaction between time and group), the right middle occipital gyrus was found with a more rapidly decreased short-range FC in PMCI. Besides, the observed decrease in this region was significantly correlated with the cognitive decline of the MCI patients in 1-year duration. These observations may underlie a relationship between the connectivity change in the right middle occipital gyrus and the disease progression in MCI individuals. Further, as previously mentioned, the rapid FC decrease in the middle occipital gyrus may also emphasize the role of visual cortex disruption in AD progression and is in line with previous evidence of impaired visual perception cortices and related dysfunction (Cronin-Golomb et al., 1995; Mandal et al., 2012; Deng et al., 2016).

The cross-sectional between-group comparison of the long-range and short-range degree revealed significantly changed connectivity in the left middle temporal gyrus and cerebellum in PMCI subjects compared with SMCI subjects. However, the observed baseline degree changes in the two regions were not included in the longitudinal results, and were not consistent with the our previous findings obtained by the FC strength analysis (Li et al., 2016). Moreover, the subsequent ROC analysis results revealed that the observed degree divergence at baseline showed limited sensitivity and specificity for predicting the occurrence of progression in MCIs. Taken together, these results may indicate that the baseline degree difference may not be a good indicator for the prognosis of MCI. However, due to the preliminary nature of this study, the sample size included was relatively small. Therefore, the prognostic utility of the cross-sectional brain FC evaluation may deserve to be further explored in studies with larger sample size and with other connectivity probes.

The major limitations of this study were the relatively short neuroimaging follow-up period and relatively small sample size. Therefore, future studies with a larger sample size, a longer follow-up period and more time points for MR acquisition are needed. Additionally, though the degree analysis using multiple correlation thresholds increases the robustness of the results, there has no widely accepted criterion for selecting the threshold range. A low threshold may lead to increased false positive rate, while high threshold may lead to the degree maps with lower sensitivity. That the reason why we didn’t further extend the upper and lower bounds of the threshold range. However, it should be noted that the selection of threshold range may have an influence to the results, and future technical investigations are encouraged to propose an optimal range.

## Conclusion

In conclusion, this study comprehensively assessed the connectivity alteration and its longitudinal changing pattern across the whole brain in PMCI patients. In the longitudinal study, compared with SMCI group, the PMCI group showed decreased long-range connectivity in the left middle occipital/supramarginal gyrus, while the short-range FC was increased in the left supplementary motor area and middle frontal gyrus and decreased in the right middle temporal pole/inferior temporal gyrus. A significant longitudinal alteration of decreased short-range degree in the right middle occipital was found in PMCI group. Taken together with previous evidence, our current findings may suggest that PMCI, compared with SMCI, might be a severe presentation of disease along the AD continuum, and the rapidly impaired connectivity in the right middle occipital gyrus may have certain indicative value for the disease progression. Furthermore, the cross-sectional degree comparison and the corresponding ROC analyses may indicate that baseline degree difference may not be a good predictor for disease progression in MCI patients. Overall, these findings provide objective evidence to characterize the alteration patterns of brain connectivity associated with disease progression in MCI patients. We hope the results will help to enrich our understanding of the neurobiological mechanism of progression in AD spectrum diseases.

## Author Contributions

YD contributes to design the work, analyze the data, and write the manuscript; KL technical support for the data analysis and revise the manuscript; LS final approval of the version to be published; DW final approval of the version to be published. YL Agreement to be accountable for all aspects of the work; PL Agreement to be accountable for all aspects of the work; KL Agreement to be accountable for all aspects of the work; WC Agreement to be accountable for all aspects of the work.

## Conflict of Interest Statement

The authors declare that the research was conducted in the absence of any commercial or financial relationships that could be construed as a potential conflict of interest.

## Footnotes

1 http://adni.loni.usc.edu/methods/documents/

2 http://www.fil.ion.ucl.ac.uk/spm/software/spm8/

3 http://adni.loni.usc.edu/methods/mri-analysis/
