# Supplementary material for: Identifying the Alteration Patterns of Brain Functional Connectivity in Progressive Mild Cognitive Impairment Patients: A Longitudinal Whole-Brain Voxel-Wise Degree Analysis
Source: Front Aging Neurosci. 2016 Aug 17;8:195. doi: 10.3389/fnagi.2016.00195 (PMC4987370; doi:10.3389/fnagi.2016.00195)
Supplement: Supplementary file 1 [file Image_1.PDF]

Supplementary figures

of

Identifying the Alternation Patterns of Brain Functional Connectivity in Progressive Mild Cognitive Impairment Patients: A Longitudinal Whole-brain Voxel-wise Degree Analysis

Yanjia Deng[a]†, Kai Liu[a]†, Lin Shi[b,c#], Yi Lei[d], Peipeng Liang[e], Kuncheng Li[e], Winnie CW Chu[a,f], Defeng Wang[a,f##], for the Alzheimer's Disease Neuroimaging Initiative*

Supplementary figure 1

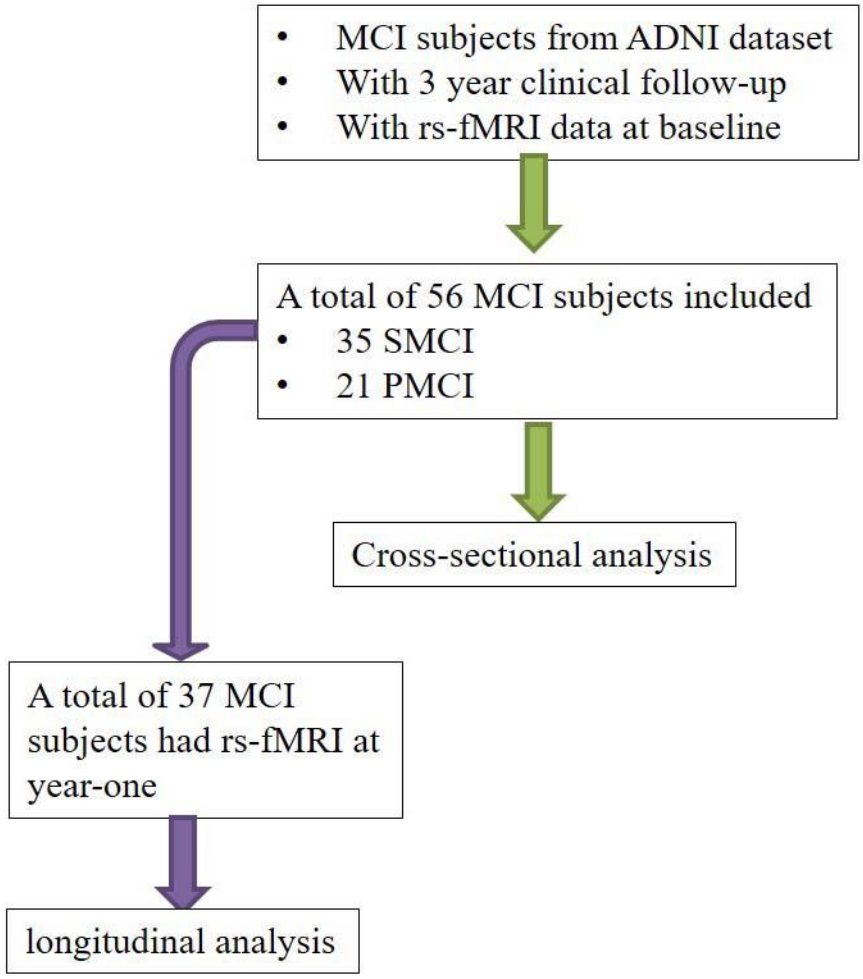

Supplementary figure 1. The flowchart of the inclusion procedures of subjects for cross-sectional and longitudinal analyses.

Supplementary figure 2

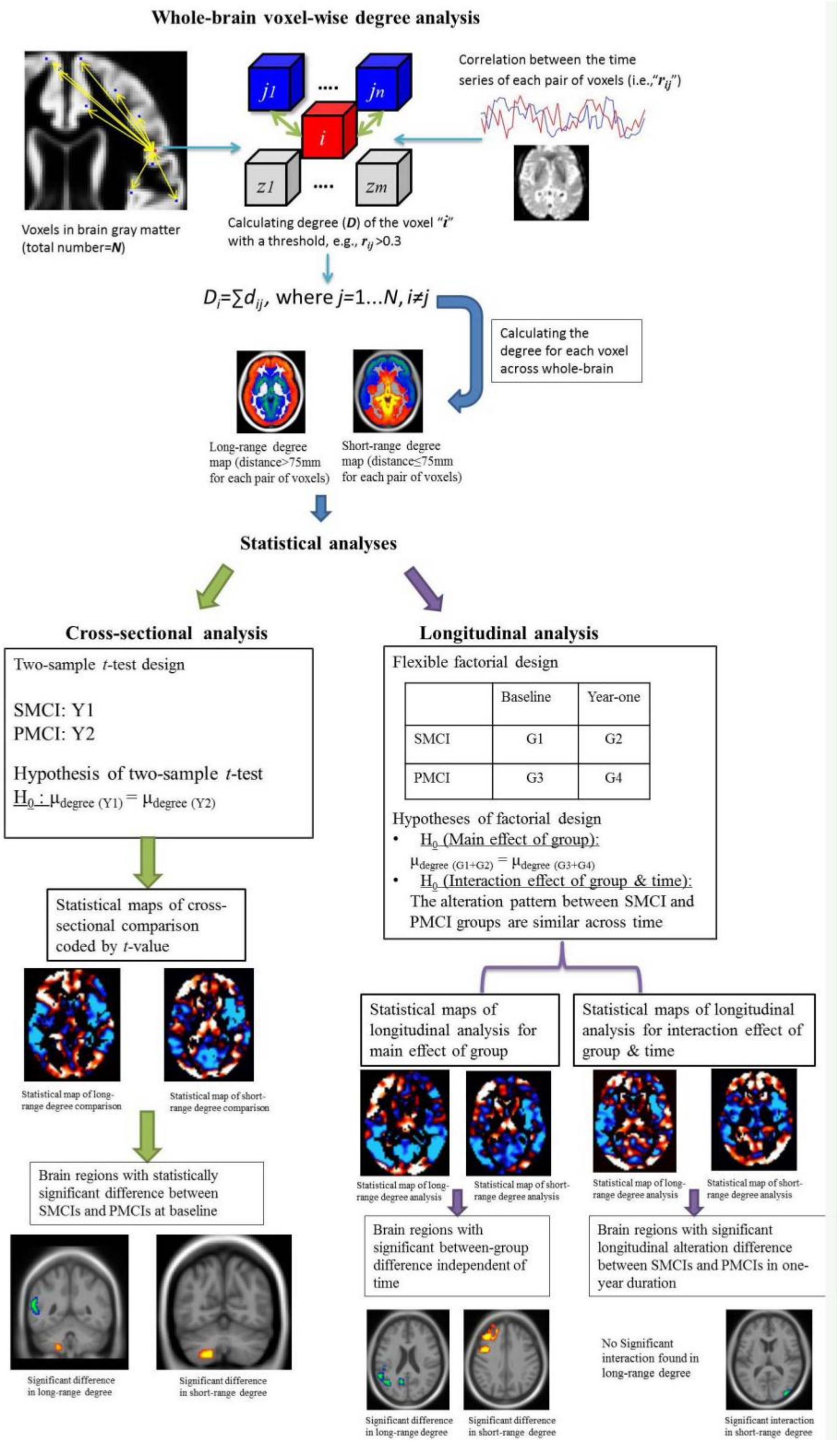

Supplementary figure 2. The flowchart of the voxel-wise degree calculation process and statistical analyses. All the analysis steps shown in the above chart are repeated at the correlation thresholds of r>0.2, 0.25, 0.3, 0.35, and 0.4. For simple and clear illustration, only the analyses and results at r>0.3 are showed in this figure as an example. SMCI: stable mild cognitive impairment; PMCI: progressive mild cognitive impairment.
