# Supplementary material for: Identifying the Alteration Patterns of Brain Functional Connectivity in Progressive Mild Cognitive Impairment Patients: A Longitudinal Whole-Brain Voxel-Wise Degree Analysis
Source: Front Aging Neurosci. 2016 Aug 17;8:195. doi: 10.3389/fnagi.2016.00195 (PMC4987370; doi:10.3389/fnagi.2016.00195)
Supplement: Supplementary file 2 [file Table_1.DOCX]

Supplementary Table 1 Demographics of subjects included for cross-sectional analyses

|  | SMCI | PMCI | *p* value |
| --- | --- | --- | --- |
| Age | 71.0±7.6 | 71.4±5.9 | 0.851 |
| Gender (M/F) | 18/17 | 11/10 | 0.945 |
| Education | 15.8±2.6 | 16.1±2.4 | 0.658 |
| mFD | 0.25 | 0.26 | 0.795 |

_PMCI: progressive mild cognitive impairment; SMCI: stable MCI._

_MMSE: Mini-Mental State Exam;_

_mFD: mean frame-wise displacement at baseline_

_Statistical level:_ *_p_*_<0.05._
